# Quality of dietary fat and risk of Alzheimer’s disease and dementia in adults aged ≥50 years: a systematic review

**DOI:** 10.29219/fnr.v66.8629

**Published:** 2022-07-28

**Authors:** Bright I. Nwaru, Jutta Dierkes, Alfons Ramel, Erik Kristoffer Arnesen, Birna Thorisdottir, Christel Lamberg-Allardt, Fredrik Söderlund, Linnea Bärebring, Agneta Åkesson

**Affiliations:** 1 Krefting Research Centre, Institute of Medicine, University of Gothenburg, Gothenburg, Sweden; 2 Centre for Nutrition, Department of Clinical Medicine, University of Bergen, Bergen, Norway; 3 Department of Laboratory Medicine and Pathology, Haukeland University Hospital, Bergen, Norway; 4 Faculty of Food Science and Nutrition, University of Iceland, Reykjavík, Iceland; 5 Department of Nutrition, Institute of Basic Medical Sciences, University of Oslo, Oslo, Norway; 6 Faculty of Sociology, Anthropology and Folkloristics & Health Science Institute, University of Iceland, Reykjavik, Iceland; 7 Department of Food and Nutrition, University of Helsinki, Helsinki, Finland; 8 Unit of Cardiovascular and Nutritional Epidemiology, Institute of Environmental Medicine, Karolinska Institutet, Stockholm, Sweden; 9 Department of Internal Medicine and Clinical Nutrition, Institute of Medicine, Sahlgrenska Academy, University of Gothenburg, Gothenburg, Sweden

**Keywords:** dietary fats, fatty acids, cognitive disorders, dementia, Alzheimer’s disease, middle age

## Abstract

**Objective:**

To identify, critically appraise, and synthesize evidence on the effect of quality of dietary fat intake and different classes of fatty acids on the risk of Alzheimer’s disease (AD) and dementia in adults aged ≥50 years.

**Methods:**

We searched MEDLINE, EMBASE, Cochrane Central of Controlled Trials, and Scopus for clinical trials and prospective cohort studies published until May 2021. Two reviewers independently screened retrieved literature, extracted relevant data, and performed risk of bias assessment. Classes of fatty acids included were saturated fatty acids (SFAs), trans fatty acids (TFAs), monounsaturated fatty acids (MUFAs), poly-unsaturated fatty acids (PUFAs), and their subtypes and sources. Given between-study heterogeneity, we did not perform meta-analyses but narratively described findings from the studies.

**Results:**

From 4,491 identified records, five articles (based on four prospective cohort studies) met the inclusion criteria. Three studies had an overall serious risk of bias, while one study had a moderate risk. Overall, we found no robust association between intake of any fatty acids type and the development of AD and dementia. For example, for SFA and TFA, there was contradictory associations reported on AD: one study found that each unit increase in energy-adjusted intake of SFA (risk ratio [RR] 0.83, 95%CI 0.70–0.98) and TFA (RR 0.80, 95%CI 0.65–0.97) was associated with a decreased risk of AD, but not dementia. For PUFA, one study found that higher quintile intake of marine-based n-3 PUFA was associated with a decreased risk of AD. The intake of other fatty acids was not associated with the outcomes. The certainty of the overall evidence was inconclusive.

**Conclusion:**

We found no clear association between the intake of various classes of fatty acids and the risk of AD and dementia in adults. More well-designed prospective studies are required to clarify these findings.

## Popular scientific summary

- Beyond genetic predisposition, lifestyle and environmental factors, like diet, are thought to play a role in the development of Alzheimer’s disease and dementia.
- The role of dietary fats has been of focus in recent decades, because of their role in inflammation.
- By performing a systematic review of previous, we found no evidence that intake of dietary fatty acids plays any important role in the development of Alzheimer’s disease and dementia in adults aged ≥50 years.

In recent decades, emerging evidence shows that the origin of Alzheimer’s disease (AD) and related dementias goes beyond genetic predisposition caused by the E4 allele of apolipoprotein E (ApoE) (1–9). Clearly, it is now understood that the pathogenesis of AD and related dementias is complex and involves an intricate interplay of modifiable environmental and lifestyle risk factors (2–9). Of the various lifestyle factors implicated, the role of diet has received increasing attention (10–13). Particularly, dietary fats have been proposed as putative risk factors in the pathogenesis of AD and related dementias (2, 14–15); however, evidence from several epidemiological and clinical studies currently remains uncertain. Certain classes of fatty acids, particularly n-3 polyunsaturated fatty acids (n-3 PUFAs), have anti-inflammatory properties (16). Given that inflammation may predispose AD and dementias (17), there has been an interest in investigating potential impact of dietary fatty acids on AD pathogenesis.

In updating the 2012 Nordic Nutrition Recommendations (NNR), the role of fat quality in the pathogenesis of AD and related dementias was one of the prioritized topics among the systematic reviews commissioned by NNR Committee. The criteria for prioritization of topics were published *a priori* (18, 19), which indicated that a new systematic review was warranted when important new scientific data have been published on a topic since the NNR 2012. Furthermore, a topic was prioritized in the case that no recent qualified systematic review exists on that topic and if the topic is of substantial health concern for Nordic or Baltic countries. Following a scoping review, the NNR Committee concluded that with an ageing population and increasing prevalence of cognitive disorders including dementia, evidence on the role of dietary fats in adverse disorders among the elderly is justified. The aim of this systematic review was to identify, critically appraise, and synthesize evidence from studies on the role of different classes of fatty acids in subsequent risk of adverse cognitive outcomes.

## Methods

The systematic review process adhered to the *a priori* published systematic review guidelines developed for the NNR 2022 (20, 21). The systematic review process also followed the recommendation of the Preferred Reporting Items for Systematic Reviews and Meta-analyses (PRISMA) (22, 23). The NNR 2022 Committee developed, using an iterative process with the authors, a focused systematic review question, which included definition of the study population, intervention/exposure, control, outcome, timeframe, study design, and settings (PI/ECOTSS) (Table 1). Our methods are detailed in a protocol registered in PROSPERO (2021: CRD42021235829).

**Table 1:** The PI/ECOTSS (population/participants, intervention/exposure, control, outcome, timeframe, study design, and settings) used to frame the systematic review question

Dietary fat quality and cognition						
Population	Intervention or exposure	Comparators	Outcomes	Timing	Setting	Study design
Adults (≥50 years)	Quality of fat (for example E% from different subtypes, such as SFA, MUFA, PUFA, PS-ratio, etc.), TFA not total amount	Other level of intake, substitution models	Outcome: specific dementias: Alzheimer’s disease (ICD8 290.10 and ICD10 F00 and G30), vascular dementia (ICD10 F01), and unspecified dementia (ICD8 290.18 and ICD10). All-cause dementia. For intervention studies: mild cognitive impairment (G31) and cognitive decline	RCTs > 1 year (intervention), cohorts minimum of 5 years follow-up	Relevant for the general population in the Nordic and Baltic countries	Prospective cohort studies, intervention studies

This study was funded by the Nordic Council of Ministers and governmental food and health authorities of Norway, Finland, Sweden, Denmark, and Iceland.

### Eligibility criteria

We included studies performed in adults aged 50 years and above from any setting relevant for the general population of the Nordic and Baltic countries (Table 1). We considered the quality of dietary fat (absolute intake in g/d adjusted for total energy intake or percent energy (E%) from saturated fatty acids [SFAs], monounsaturated fatty acids [MUFAs], polyunsaturated fatty acids [PUFAs], PUFA:SFA ratio (PS-ratio), and trans fatty acids [TFAs]). As the interest of this review was on the effects of the intake of nutrients, studies on sources of fatty acids, e.g. olive oil, were excluded. Total amount of fat was considered as a confounder but not as exposure by itself. The study designs of interest were randomized (RCT) or non-randomized intervention trials, prospective cohort studies, nested case–control studies, and case–cohort studies. These study designs were included to ensure that the evidence informing the NNR recommendation is derived from studies with most robust design. For observational studies in particular, only designs that are inherently prospective in nature were considered. Intervention trials must have had at least 1 year of intervention, while observational studies must have had at least 5 years of follow-up to be included. A longer, as opposed to shorter follow-up, is generally beneficial, since diseases develop over time. Nevertheless, the field of research addressing fat quality and cognition is still evolving, and it has not been established how long it takes to detect the effects of a given exposure. Moreover, different sub-outcomes within cognition can also have different rates of disease development, where some develop faster than others. The time periods chosen for this systematic review are a tradeoff between being inclusive, and thereby taking the risk of including articles where the intervention or exposure was too short to have any appreciable effect, to instead capture fewer articles but with a higher possibility to detect an ‘effect’ if there is one. The discrepancy between cohort studies and intervention studies was the consequence of intervention studies more difficult to implement over long periods. In addition, it is likely that the baseline risk differs between observational and intervention studies due to differences in age. A minimum of 5 years of follow-up in a cohort study, therefore, appears reasonable. For intervention studies, studies were included if the intervention was compared to usual diet, in the absence of dietary advice or nutrient supplementation, or placebo/other comparators used. In cohort studies, comparison was made to lower intake (e.g. quantiles), or other level of intake or substitution models (substituting e.g. SFA with MUFA or PUFA). We examined the effect of quality of fat on adverse cognitive disorders classified in the following categories: AD, vascular dementia, unspecified dementia, all-cause dementia, and AD mortality. For RCT studies, mild cognitive impairment and cognitive decline were also eligible as outcomes.

### Information sources and search strategy

A comprehensive search strategy of MEDLINE, EMBASE, Cochrane Central of Controlled Trials, and Scopus was made by research librarians at Karolinska Institutet University Library, and peer reviewed by the University of Oslo Library of Medicine and Science, up to May 2021. The search strategy (Supplementary file 1) was developed in collaboration with the authors, led by BN and JD. There were no exclusions by publication date or language. The reference list of included studies was also screened to identify potentially eligible studies. We did not include the gray literature because of its uncertain methodological quality (20, 21).

### Selection and data collection processes

All literature retrieved from the database searches were exported to Endnote, where duplicate reports were removed, after which the remaining papers were exported to Rayyan, where the literature screening was performed. The literature screening was performed by two members (AR and EA) of the team, working independently. Literature screening was first piloted with approximately 10% of the obtained titles and abstracts before full literature screening on the remaining 90% of the papers. If at least one of the assessors voted for inclusion, the paper was included in the full text screening. Potentially eligible papers were retrieved and read in full text by the two reviewers. Discrepancies between assessors were resolved by discussion or arbitration by a senior member of the research team (AÅ).

We developed and piloted a data extraction form, which was used to extract relevant data from the full-text papers by three reviewers working independently (CLA, BT, and FS). Any discrepancies in the data extraction were resolved by discussion. The data extraction form included a minimum of the following items: the full reference, eligibility, methods, participants and settings; interventions/exposures; outcomes; main results; and confounding variables. Nutrition-specific elements, such as intake levels/doses, food sources, method for dietary assessment, validation of the dietary assessment method, food composition database used, and assessment of nutrition status, were also extracted. The study is the unit of interest; duplicate papers from the same study were extracted as one study, where relevant.

### Study risk of bias assessment

Two reviewers (BN and LB) independently evaluated the risk of bias in all included studies. Any discrepancies were resolved by discussion. Since no RCTs were retrieved, assessment of risk of biased was based on the ‘Risk of Bias for Nutrition Observational Studies’ (RoB-NObS) tool (developed by the USDA’s Nutrition Evidence Systematic Review [NESR]) alone (24), which is partly based on the Risk of Bias in Non-randomized Studies of Intervention (ROBINS-I) instrument (25). The risk of bias in each individual study was classified as low risk, moderate, serious, or critical, both at each domain of bias assessment and overall. The details for considerations for grading of each domain of the study and overall grading are provided in the RoB-NObS document, and it should be noted that a study is judged to be at high risk of bias overall if one of its domains has a high risk of bias grading.

### Synthesis methods

We performed a narrative synthesis of the included studies by describing the characteristics and context of the studies, their strengths and limitations, heterogeneity (in study characteristics and results), and relevance. Following the recommendations of the Agency for Healthcare Research and Quality (AHRQ) and the Cochrane Handbook, our a priori criteria to perform meta-analysis stipulated that more than three independent RCTs or five cohort studies must be available on each specific question for a meta-analysis to be undertaken (26–28). In addition to not meeting these conditions given fewer studies, high heterogeneity between the included studies did not allow us to perform any meta-analysis.

### Certainty assessment

Strength of evidence was categorized according to the World Cancer Research Fund’s grading: ‘Convincing’, ‘Probable’, ‘Limited – suggestive’, ‘Limited – no conclusion’, ‘Substantial effects unlikely’ (18, 20). The quality (risk of bias), quantity, consistency, and precision in the body of evidence were used for categorizing the strength of the evidence. A *convincing* body of evidence was established as strong enough to support a causal relationship or lack of a relationship in which several conditions are met, including evidence coming from more than one study type. A *probable* body of evidence was supported when strong enough to support a probable causal relationship, and there was evidence from at least two independent cohort studies, no unexplained heterogeneity between or within study types, good-quality studies to confidentially exclude possible random or systematic errors, and evidence for biological plausibility. A *limited – suggestive* was supported when there was evidence from at least two independent cohort studies: a consistent direction of effect and evidence for biological plausibility. A *limited – no conclusion* evidence was established if the evidence is so limited that no firm conclusion could be made. An evidence strong enough to support a convincing absence of a causal relationship was considered *substantial effects unlikely*.

## Results

### Study selection search results

**Fig. 1:**
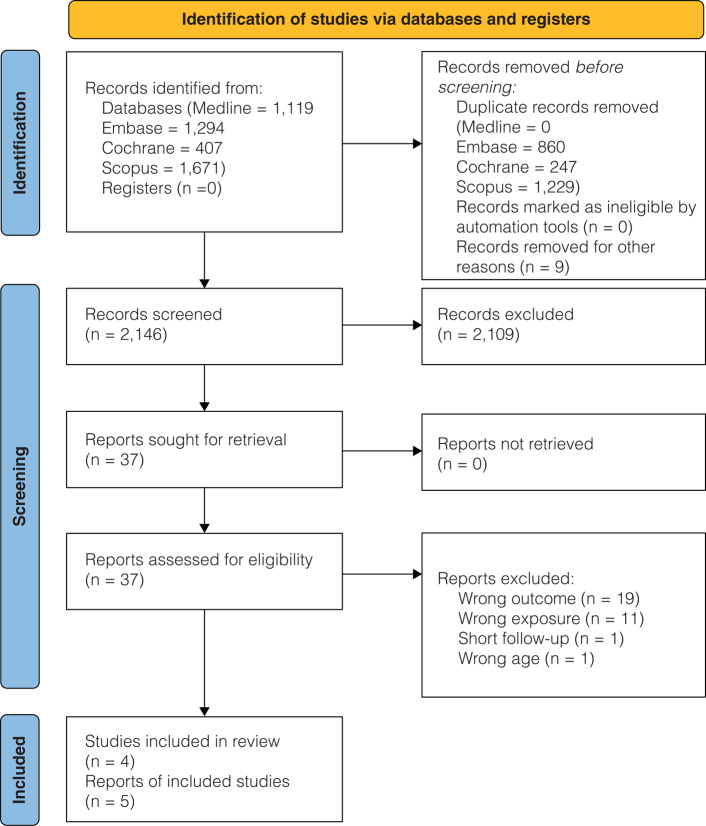
PRISMA flow diagram for database searches and study screening.

A total of 2,146 records were retrieved from the database searches after de-duplication; of which 2,109 were excluded after title and/or abstract screening. Of the 37 full-text papers evaluated, five met the criteria to be included in the review (originating from four individual studies) (29–33). Figure 1 gives the flowchart for the literature screening. Reasons for excluding each of the remaining 32 studies after full-text screening are included in Supplementary file 2.

### Study characteristics

In total, 530,576 participants were analyzed across the four included independent studies, with mean age range at baseline of 63–76 years. The participants were mainly healthy adults recruited from the general population or community. All the four studies were prospective cohort studies. Two of the studies were conducted in the USA (30, 33), one in Finland (31, 32), and one in the Netherlands (29). Fatty acids and their different classes were evaluated in all the studies. All studies assessed intake of the fatty acids using semi-quantitative food frequency questionnaires. All included studies studied SFA, MUFA, and PUFA, while only two studies considered TFA (29, 33). Fat intake was measured as absolute intake (g/d) with adjustment for total energy intake in the regression analyses (Engelhart, Gustafson, Laitinen) or as E% (Zhuang). Both AD and dementia were investigated across all studies, assessed using internationally recommended standard measurements in all studies, often using relevant ICD codes.

### Risk of bias in included studies

Of the four included studies, three received an overall serious risk of bias grading (29–32), while one study received a moderate risk of bias grading (33). All studies were graded as at low risk of bias for outcome assessment, which was the component with highest grading. Three of the four studies received serious risk of bias for confounding (29–32), which was the component with the worst grading. Grading for participant selection, exposure classification, and missing data was moderate for all, but one study (Table 2).

**Table 2:** Details of risk of bias results for included studies

Study, source of funding*	Country	RoB-NObs results for prospective cohort studies							
		Confounding	Selection	Exposure classification	Departures from intended exposures	Missing data	Outcome measurement	Selection of reported result	Overall bias
Engelhart et al. 2002; public funding	Netherlands	Serious risk	Moderate risk	Moderate risk	Low risk	Moderate risk	Low risk	Low risk	Serious risk
Gustafson et al. 2020; public funding	USA	Serious risk	Moderate risk	Moderate risk	Serious risk	Moderate risk	Low risk	Low risk	Serious risk
Kivipelto et al. 2008 and Laitinen et al. 2006; public funding	Finland	Serious risk	Moderate risk	Serious risk	Serious risk	Moderate risk	Low risk	Moderate risk	Serious risk
Zhuang et al. 2019; public funding	USA	Moderate risk	Moderate risk	Moderate risk	Low risk	Moderate risk	Low risk	Low risk	Moderate risk

* Sources of funding were categorized into public and private sources. Public sources include governmental institutions and charities, while private sources included non-governmental private companies and industries.

### Association between SFA intake and risk of AD and dementia

Overall, most studies showed no putative association between SFA intake and risk of AD and dementia, except for the studies by Engelhart et al. and Kivipelto et al. (Table 3). Engelhart et al. showed that one standard deviation (SD) increment in energy-adjusted intake of SFA was associated with an increased risk of developing AD. In contrast, the study by Kivipelto et al., only the second and not the third and fourth quartiles, compared to the first, of dietary SFA intake was statistically significantly associated with an increased risk of developing dementia and AD (Table 3). Zhuang et al. further found no significant associations of isocaloric substitution of SFA with other classes of fatty acids with the risk of AD mortality (Supplementary file 3).

**Table 3:** Characteristics and results of studies on saturated fatty acids (SFAs) and adverse cognitive outcomes in adults ≥50 years of age

Study, country	Study design	Participants		Intervention/exposure and assessment	Outcome and assessment	Estimates for the association between SFA and outcomes
		Sampling method, source	Recruited/number analyzed, age			
Engelhart et al. 2002, Netherlands	Prospective cohort study	Healthy adult general population	7,983/5,395 (3,183 women; 2,212 men); mean age (SD): 67.7 (7.8)	Assessed using semiquantitative food frequency questionnaire (FFQ)	Incident dementia (vascular dementia and other types of dementia) and Alzheimer’s disease (AD). Dementia diagnosed following the criteria of the Diagnostic and Statistical Manual of Mental Disorders. AD diagnosed following the criteria of the National Institute of Neurological and Communication Disorders and Stroke	Results are given as per SD increase in the intake of energy-adjusted fat. Total dementia: rate ratio (RR) 0.91 (95%CI 0.79–1.05); AD: RR 0.83 (95%CI 0.70–0.98); vascular dementia: RR 1.03 (95%CI 0.73–1.46)
Gustafson et al. 2020, USA	Prospective cohort study	General, multiethnic population	2,647/2,612 (1,761 women; 851 men); mean age (SD): 76.3 (6.4)	Assessed using 61-item semiquantitative FFQ, adapted from the Harvard FFQ	AD, assessed following the criteria of the Blessed Dementia Rating Scale, the Schwab and England Activities of Daily Living Scale	1st tertile (reference): hazard ratio (HR) for AD: 2nd tertile: HR 0.82 (95%CI 0.62–1.09); 3rd tertile: HR 1.25 (95%CI 0.88–1.77)
Kivipelto et al. 2008 and Laitinen et al. 2006, Finland	Prospective cohort study	Healthy adult general population	2,000/1,449 (900 women; 549 men); mean age (SD): midlife exam. 50.4 (6.0); 71.3 (4.0)	Assessed using self-administered semiquantitative FFQ	Dementia, assessed following the criteria of the Diagnostic and Statistical Manual of Mental Disorders (4th edition)	1st quartile (reference): odds ratio (OR). Dementia: 2nd quartile: OR 2.45 (95%CI 1.10–5.47); 3rd quartile: OR 1.39 (95%CI 0.53–3.69); 4th quartile: OR 2.74 (95%CI 0.65–11.56). AD: 2nd quartile: OR 3.82 (95%CI 1.48–9.87); 3rd quartile: OR 1.90 (95%CI 0.63–5.71); 4th quartile: OR 2.34 (95%CI 0.51–10.74)
Zhuang et al 2019, USA	Prospective cohort study	General population	567,169/521,120 (306,365 men and 214,755 women); mean age 62.75	124-item FFQ, developed as the Diet History Questionnaire at National Cancer Institute	AD, based on ICD-9 codes: (331) and ICD-10 (G30)	1st quintile (reference): HR for AD: 2nd quintile: HR 0.99 (95%CI 0.85–1.16); 3rd quintile: HR 1.11 (95%CI 0.93–1.33); 4th quintile: HR 1.06 (95%CI 0.87–1.29); 5th quintile: HR 1.14 (95%CI 0.92–1.42)

### Association between MUFA intake and risk of AD and dementia

There were no statistically significant associations between the intake of MUFA and the risk of AD or dementia in any of the four included studies (Table 4). The role of plant- and animal-based MUFA for AD mortality was further considered by Zhuang et al., but neither of these sources of MUFA were statistically significantly associated with the outcomes (Supplementary file 3).

**Table 4:** Characteristics and results of studies on monounsaturated fatty acids (MUFAs) and adverse cognitive outcomes in adults ≥ 50 years of age

Study, country	Study design	Participants		Intervention/exposure and assessment	Outcome and assessment	Estimates for the association between MUFA and outcomes
		Sampling method, source	Recruited/number analyzed, age			
Engelhart et al. 2002, Netherlands	Prospective cohort study	Healthy adult general population	7,983/5,395; mean age (SD): 67.7 (7.8)	Assessed using semiquantitative food frequency questionnaire (FFQ)	Incident dementia (vascular dementia and other types of dementia) and Alzheimer’s disease (AD). Dementia diagnosed following the criteria of the Diagnostic and Statistical Manual of Mental Disorders. AD diagnosed following the criteria of the National Institute of Neurological and Communication Disorders and Stroke	Results are given as per SD increase in the intake of energy-adjusted fat. Total dementia: rate ratio (RR) 0.96 (95%CI 0.84–1.10); AD: RR 0.91 (95%CI 0.79–1.07); vascular dementia: RR 1.05 (95%CI 0.76–1.47)
Gustafson et al. 2020, USA	Prospective cohort study	General, multiethnic population	2,647/2,612; mean age (SD): 76.3 (6.4)	Assessed using 61-item semiquantitative FFQ, adapted from the Harvard FFQ	AD, assessed following the criteria of the Blessed Dementia Rating Scale, the Schwab and England Activities of Daily Living Scale	1st tertile (reference): hazard ratio (HR) for AD: 2nd tertile: HR 0.97 (95%CI 0.73–1.29); 3rd tertile: HR 1.42 (95%CI 0.99–2.05)
Kivipelto et al. 2008 and Laitinen et al. 2006, Finland	Prospective cohort study	Healthy adult general population	2,000/1,449; mean age (SD): midlife exam. 50.4 (6.0); 71.3 (4.0)	Assessed using self-administered semiquantitative FFQ	Dementia, assessed following the criteria of the Diagnostic and Statistical Manual of Mental Disorders (4th edition)	1st quartile (reference): Odds ratio (OR). Dementia: 2nd quartile: OR 0.49 (95%CI 0.21–1.13); 3rd quartile: OR 0.83 (95%CI 0.36–1.92); 4th quartile: OR 1.01 (95%CI 0.29–3.55). AD: 2nd quartile: OR 0.58 (95%CI 0.23–1.46); 3rd quartile: OR 1.03 (95%CI 0.41–2.61); 4th quartile: OR 1.02 (95%CI 0.26–4.01)
Zhuang et al 2019, USA	Prospective cohort study	General population	567,169/521,120; mean age 62.75	124-item FFQ, developed as the Diet History Questionnaire at National Cancer Institute	AD, based on ICD-9 codes: (331) and ICD-10 (G30)	1st quintile (reference): HR for AD: 2nd quintile: HR 0.93 (95%CI 0.78–1.11); 3rd quintile: HR 0.90 (95%CI 0.72–1.12); 4th quintile: HR 0.83 (95%CI 0.64–1.07); 5th quintile: HR 0.85 (95%CI 0.63–1.15)

### Association between PUFA intake and the risk of AD and dementia

With one exception, the studies showed no putative association between total PUFA, n-3 PUFA, or n-6 PUFA and the risk of developing AD and dementia. Kivipelto et al. found that only the second versus first quartile of the intake of total PUFA was associated with a decreased risk of developing dementia (Table 5). Although the results were statistically non-significant, there was no consistent association between PUFA intake and the outcomes: for instance, in the studies by Engelhart et al. and Zhuang et al., higher intake of total PUFA, n-3 PUFA, and n-6 PUFA showed a small increased risk of developing AD and dementia. However, in the studies by Gustafson et al. and Kivipelto et al., higher intake of total PUFA tended to be associated with a decreased risk of AD and dementia (Table 5). The study by Zhuang et al. further considered the role of subtypes of n-3 PUFA and n-6 PUFA (e.g. α-linolenic, linoleic, and arachidonic acids), but none of these was statistically significantly associated with the outcomes (Supplementary file 3). However, increasing quintile intake of marine-based n-3 PUFA was associated with a decrease of AD mortality in the study by Zhuang et al. (Supplementary file 3).

**Table 5:** Characteristics and results of studies on polyunsaturated fatty acids (PUFAs)a and adverse cognitive outcomes in adults ≥50 years of age

Study, country	Study design	Participants		Intervention/exposure and assessment	Outcome and assessment	Estimates for the association between PUFAs and outcomes
		Sampling method, source	Recruited/number analyzed, age			
Engelhart et al. 2002, Netherlands	Prospective cohort study	Healthy adult general population	7,983/5,395; mean age (SD): 67.7 (7.8)	Assessed using semiquantitative food frequency questionnaire (FFQ)	Incident dementia (vascular dementia and other types of dementia) and Alzheimer’s disease (AD). Dementia diagnosed following the criteria of the Diagnostic and Statistical Manual of Mental Disorders. AD diagnosed following the criteria of the National Institute of Neurological and Communication Disorders and Stroke	Results are given as per SD increase in the intake of energy-adjusted fat. Rate ratio (RR): total dementia: PUFA 1.05 (95%CI 0.80–1.38); N-6 PUFA 1.03 (95%CI 0.77–1.36); N-3 PUFA 1.07 (95%CI 0.94–1.22). AD: PUFA 1.09 (95%CI 0.79–1.50); N-6 PUFA 1.07 (95%CI 0.77–1.49); N-3 PUFA 1.07 (95%CI 0.91–1.25); vascular dementia: PUFA 1.16 (95%CI 0.58–2.33); N-6 PUFA 1.09 (95%CI 0.52–2.26); N-3 PUFA 1.17 (95%CI 0.85–1.59)
Gustafson et al. 2020, USA	Prospective cohort study	General, multiethnic population	2,647/2,612; mean age (SD): 76.3 (6.4)	Assessed using 61-item semiquantitative FFQ, adapted from the Harvard FFQ	AD, assessed following the criteria of the Blessed Dementia Rating Scale, the Schwab and England Activities of Daily Living Scale	1st tertile (reference): hazard ratio (HR) for association between PUFA and AD: 2nd tertile: HR 0.77 (95%CI 0.59–1.02); 3rd tertile: HR 0.76 (95%CI 0.55–1.07)
Kivipelto et al. 2008 and Laitinen et al. 2006, Finland	Prospective cohort study	Healthy adult general population	2,000/1,449; mean age (SD): midlife exam. 50.4 (6.0); 71.3 (4.0)	Assessed using self-administered semiquantitative FFQ	Dementia, assessed following the criteria of the Diagnostic and Statistical Manual of Mental Disorders (4th edition)	1st quartile (reference): Odds ratio (OR) for PUFA. Dementia: 2nd quartile: OR 0.40 (95%CI 0.17–0.94); 3rd quartile: OR 0.67 (95%CI 0.29–1.56); 4th quartile: OR 0.48 (95%CI 0.16–1.38). AD: 2nd quartile: OR 0.53 (95%CI 0.21–1.37); 3rd quartile: OR 0.70 (95%CI 0.27–1.85); 4th quartile: OR 0.69 (95%CI 0.22–2.19)
Zhuang et al. 2019, USA	Prospective cohort study	General population	567,169/521,120; mean age 62.75	124-item FFQ, developed as the Diet History Questionnaire at National Cancer Institute	AD, based on ICD-9 codes: (331) and ICD-10 (G30)	1st quintile (reference): HR for AD. PUFA: 2nd quintile: HR 1.04 (95%CI 0.90–1.21); 3rd quintile: HR 1.04 (95%CI 0.88–1.22); 4th quintile: HR 1.02 (95%CI 0.86–1.21); 5th quintile: HR 0.96 (95%CI 0.79–1.17). N-6 PUFA 2nd quintile: HR 1.06 (95%CI 0.90–1.24); 3rd quintile: HR 1.05 (95%CI 0.88–1.27); 4th quintile: HR 1.05 (95%CI 0.85–1.30); 5th quintile: HR 1.00 (95%CI 0.78–1.29). N-3 PUFA 2nd quintile: HR 1.05 (95%CI 0.91–1.21); 3rd quintile: HR 0.90 (95%CI 0.77–1.06); 4th quintile: HR 0.95 (95%CI 0.79–1.13); 5th quintile: HR 0.83 (95%CI 0.68–1.02)

^a^Only results for total PUFA, N-6, and N-3 PUFAs are given in the table. Results for other types of PUFA, including animal- and plant-based PUFAs, are given in the Supplementary file.

### Association between TFA intake and the risk of AD and dementia

In the study by Engelhart et al., one SD-increment in the intake of energy-adjusted TFA was associated with a reduced risk of developing AD (20% reduced risk, 95% CI 3–35%) but not with total or vascular dementia. However, higher quintiles of TFA intake were not statistically significantly associated with AD mortality in the study by Zhuang et al. (Table 6).

**Table 6:** Characteristics and results of studies on trans fatty acids (TFAs) and adverse cognitive outcomes in adults ≥50 years of age

Study, country	Study design	Participants		Intervention/exposure and assessment	Outcome and assessment	Estimates for the association between TFA and outcomes
		Sampling method, source	Recruited/number analyzed, age			
Engelhart et al. 2002, Netherlands	Prospective cohort study	Healthy adult general population	7,983/5,395; mean age (SD): 67.7 (7.8)	Assessed using semiquantitative food frequency questionnaire (FFQ)	Incident dementia (vascular dementia and other types of dementia) and Alzheimer’s disease (AD). Dementia diagnosed following the criteria of the Diagnostic and Statistical Manual of Mental Disorders. AD diagnosed following the criteria of the National Institute of Neurological and Communication Disorders and Stroke	Results are given as per SD increase in intake of energy-adjusted fat. Total dementia: rate ratio (RR) 0.90 (95%CI 0.77–1.06); AD: RR 0.80 (95%CI 0.65–0.97); vascular dementia: RR 1.01 (95%CI 0.71–1.44)
Zhuang et al. 2019, USA	Prospective cohort study	General population	567,169/521,120; mean age 62.75	124-item FFQ, developed as the Diet History Questionnaire at National Cancer Institute	AD, based on ICD-9 codes: (331) and ICD-10 (G30)	1st quintile (reference): HR for AD. 2nd quintile: HR 0.94 (95%CI 0.81–1.09); 3rd quintile: HR 0.97 (95%CI 0.82–1.14); 4th quintile: HR 0.99 (95%CI 0.83–1.18); 5th quintile: HR 1.13 (95%CI 0.94–1.36)

### Certainty in the evidence

#### SFA and adverse cognitive outcomes

On the association between SFA and the development of AD and dementia, given the contradictory evidence and the high risk of bias, we considered the certainty of this evidence as *limited – no conclusion*.

#### MUFA and adverse cognitive outcomes

Given the absence of any statistically significant association between MUFA and AD and dementia, we considered this evidence as *limited – no conclusion*, due to tendencies toward contradictory findings.

#### PUFA and adverse cognitive outcomes

Of the four studies included, two showed a trend, generally statistically non-significant, toward an increased risk of AD and dementia with the intake of PUFA, while two showed the opposite effect. On the basis of contradictory findings, we considered the certainty of this evidence as *limited – no conclusion*.

#### TFA and adverse cognitive outcomes

Of the two studies that investigated TFA, one showed a significantly reduced risk between TFA intake and AD, but one showed a statistically non-significant tendency toward a decreased risk. We considered the certainty of this evidence as *limited – no conclusion*. On the association between TFA and the development of dementia, only one study considered this question, but found no statistically significant effect; hence, we considered this evidence as *limited – no conclusion*, since there was no basis to support a convincing presence or absence of a causal relationship.

## Discussion

### Summary of key findings

Very few studies were identified fulfilling the eligibility criteria. Overall, we found no robust association between the intake of any fatty acid type and the development of dementia or AD. For SFA and TFA, there was even contradictory associations reported on specifically AD. There was no clear association between MUFA and PUFA and their subtypes, and AD and dementia. In the light of these findings, we considered the overall evidence on the role of fatty acids in the development AD and dementia to be at best inconclusively limited.

### Strengths and limitations

We followed established processes for undertaking robust systematic reviews. The NNR 2022 Committee, a priori, established criteria for the prioritization and selection of a systematic review topic (20, 21). To enhance transparency in the review process, we developed and registered a detailed protocol prior to undertaking the review. To identify relevant studies on the review topic, we searched four leading electronic databases, which cover the majority of the literature in medicine and public health. In addition to this comprehensive database searches, we made no language restriction. It is, therefore, unlikely that we missed any relevant literature to the review topic. Furthermore, the review processes were rigorously implemented, with independent assessments taken at each stage, including literature screening and data extraction. While our prior intention was to include studies on quality of dietary fat as expressed as E%, we included also studies with other units, particularly g/d as E% was not always used by all studies.

Three of the four included studies were graded as having serious risk of bias (29–32), which may constitute a limitation of the underlying evidence. The majority of the studies were prone to some of the limitations inherent in many observational epidemiologic studies, including particularly the possibilities of inadequate adjustment for confounding factors, thereby given a possibility for residual/unmeasured confounding across the reported estimates in the studies. Consequently, the proportion of the effect estimates due in part by the result of residual confounding or due to actual causal impact of fatty acids on the outcomes is uncertain. Heterogeneity between the studies in the study processes and in definition of the intake of fatty acids prevented undertaking meta-analysis to derive pooled effect estimates. The very limited outcomes that were eligible and the short follow-up times could constitute further limitation to this study.

### Comparison to previous studies

Two previous systematic reviews reported mixed results on the association between the intake of dietary fats and the risk of cognitive outcomes. In the study by Barnard et al., who included also two of the studies included in the present review, the association between SFA and TFA and dementia was synthesized in prospective cohort studies (34). The authors included four relevant studies that met set inclusion criteria but found that the studies reported contradictory results. Similar to our study, that study failed to perform a meta-analysis as a result of heterogeneity between included studies (34). In the second study, Cao et al. performed a systematic review and meta-analysis to evaluate the association between dietary fat intake (total fat, SFA, MUFA, and PUFA) and cognitive function among adults aged ≥55 years old (35). They included nine studies (including Laitinen 2006); of these, six with follow-up <5 years, and on the basis of meta-analyses, they found that SFA was associated with an increased risk of cognitive impairment and AD. Overall, they found significant heterogeneity between the studies that evaluated the association between MUFA and cognitive outcomes (35). Although we were unable to perform meta-analyses due heterogeneity between the studies, our findings in general were in line with those reported by Cao et al that performed meta-analyses (35). The slight differences observed, particularly for MUFAs and PUFAs, could potentially be due to the slight age differences between the current review (≥50 years) and Cao et al.’s review (≥55 years).

### Interpretation and implications of findings

Advances in our understanding of the disease mechanisms for AD and related dementias show that they are diseases with complex pathogenesis, involving a complex interplay of genetic, environmental, and lifestyle risk factors. The dietary fat quality, especially a higher intake of SFA/TFA, has been proposed to be linked with risk of dementia, including AD (32). Potential mechanisms may include the elevation of cholesterol (36, 37). When discussing the effect of dietary fat on blood cholesterol levels, it has to be taken into account which other macronutrient is replaced – whether carbohydrates or other types of fats are replaced, as the effect will be different (36, 38). TFA appears to decrease the levels of high-density lipoprotein and increase the levels of low-density lipoprotein, has pro-inflammatory effects, and increases the risk of insulin resistance and coronary heart disease (36). Also, substitution of saturated fats with PUFA and MUFA may lower low-density lipoprotein cholesterol levels and may increase high-density lipoprotein cholesterol levels (37). Dietary fatty acids have further been associated with inflammation, obesity, insulin sensitivity, and hemostasis, and most of these have also a link to the development of dementia/cognitive decline (38). Based on the current systematic review, we were neither able to corroborate the hypothesized role of SFA/TFA in the development of AD or dementia nor able to support any beneficial associations between MUFA/PUFA and adverse cognitive outcomes. Obviously, there is a need for high-quality studies, but on the other hand, it must be emphasized that fats occur as complex mixtures in the diet, and the assessment of particularly its subtypes is challenging. Both the source of the dietary fats and the overall dietary patterns in the study population may have implications for the assessment as well as the interpretation of the results. One example is a dietary pattern high in animal fats such as meat and dairy, which often is the major source of SFA. Yet, these foods often contain plenty of MUFA, which implies that a diet high in MUFA could consist of foods either high in animal fats or in plant oils, or a mix thereof. Investigation of the dietary pattern that also takes into account other nutrients provided by the overall diet would, thus, be required. An additional challenge is the dietary assessment, which is usually done once at baseline, and the long-term follow-up, which does not allow us to adjust for any dietary changes that may occur. This is especially important in the case of TFA, as the intake has declined substantially due to changes of processing in the food industry (39). In future studies, great effort is required in disentangling the effect of confounding factors on the observed associations. In all but one of the included studies, quality grading for adjustment of confounding factors was ‘Serious’. This signaled inadequate identification of potential confounding factors that should necessarily be controlled in the analyses, leading to over- or under-adjustment of confounding in the analysis. The complex nature of diet-disease association requires robust processes in identification and adjustment of confounding factors. While this is not a trivial undertaking, contemporary approaches, such as directed acyclic graphs (DAGs), have become useful in many contexts of observational studies, thus should become necessary tool for future studies.

## Conclusions

We found no putative association between the intake of various classes of fatty acids and their subtypes and the development of adverse cognitive outcomes in adults. If any potential effect exists, we consider such at best inconclusively limited. More long-term prospective cohort studies may help to clarify and elaborate on these observations.

## Funding

Funding was received from the Nordic Council of Ministers and governmental food and health authorities of Norway, Finland, Sweden, Denmark, and Iceland.

## Registration

Prospero registration number CRD42021235829.

## Supplementary Material

Quality of dietary fat and risk of Alzheimer’s disease and dementia in adults aged ≥50 years: a systematic review
