# Supplementary material for: Quality of dietary fat and risk of Alzheimer’s disease and dementia in adults aged ≥50 years: a systematic review
Source: Food Nutr Res. 2022 Jul 28;66:10.29219/fnr.v66.8629. doi: 10.29219/fnr.v66.8629 (PMC9338447; doi:10.29219/fnr.v66.8629)
Supplement: Quality of dietary fat and risk of Alzheimer’s disease and dementia in adults aged ≥50 years: a systematic review [file FNR-66-8629-s001.zip › 8629-Supplementary Material-File-1.docx]

Supplementary File 1

Search Strategies

Medline

| **#** | **Searches** |
| --- | --- |
| 1 | exp Dietary Fats/ |
| 2 | Fatty Acids/ or Fatty Acids, Essential/ or Fatty Acids, Monounsaturated/ or Fatty Acids, Unsaturated/ or Trans Fatty Acids/ |
| 3 | exp Fatty Acids, Omega-3/ or exp Fatty Acids, Omega-6/ |
| 4 | 8,11,14-Eicosatrienoic Acid/ or Arachidonic Acid/ or Caproates/ or Caprylates/ or Decanoic Acids/ or Eicosanoic Acids/ or Lauric Acids/ or Myristic Acid/ or Oleic Acid/ or Palmitic Acid/ or Plant Oils/ or Stearic Acids/ |
| 5 | (fatty acid* adj3 (long chain or medium chain or n3 or n-3 or n6 or n-6 or odd chain or omega-3 or omega-6)).ti,ab,kf. |
| 6 | (fatty acid* and (consum* or diet* or intake or ingest*)).ti,ab,kf. |
| 7 | (butter or dietary fat* or dressing* or ghee or lard or margarine).ti,ab,kf. |
| 8 | ((fatty acid* or fat or fats) adj3 (conjugated or essential or monounsaturat* or polyunsaturat* or saturat* or trans or unsaturat*)).ti,ab,kf. |
| 9 | (dha or epa or mufa or lc-pufa or pufa or sfa or tfa or transfat*).ti,ab,kf. |
| 10 | (oil? and (consum* or diet* or intake or ingest*)).ti,ab,kf. |
| 11 | (8,11,14-Eicosatrienoic acid or alpha-Linolenic acid or arachidic acid or arachidonic acid or behenic acid or caproates or capric acids or caprylates or decanoic acids or cervonic acid or cerotic acid or dihomo-gamma-linolenic acid or docosahexaenoic acid or docosanoic acid or eicosanoic acids or eicosapentaneoic acid or elaic acid or gamma-linolenic acid or gondoic acid or heptadecanoic acid or hexacosanoic acid or icosapentaenoic acid or lauric acids or lignoceric acid or linoleic acid or linolelaidic acid or margaric acid or montanic acid or myristic acid or octanoic acid or oleic acid or palmitoleic acid or palmitole* acid or palmitic acid or paullinic acid or pentadecanoic acid or pentadecylic acid or stearic acids or stearidonic acid).ti,ab,kf. |
| 12 | or/1-11 |
| 13 | Cognition Disorders/ or Cognitive Dysfunction/ or exp Dementia/ |
| 14 | (Alzheimer* or aphasia or Binswanger* or CADASIL or CADASILM or dementia* or frontotemporal lobar degeneration).ti,ab,kf. |
| 15 | (cognit* adj2 (decline or disorder* or dysfunction* or impair*)).ti,ab,kf. |
| 16 | (subcortical adj3 (encephalopathy or leukoencephalopathy)).ti,ab,kf. |
| 17 | or/13-16 |
| 18 | 12 and 17 |
| 19 | Middle Aged/ or exp Aged/ |
| 20 | (aged or aging or ageing or elder* or geriatr* or sexagenarian* or septuagenarian* or octogenarian* or nonagenarian* or centenarian* or middle* age* or old age or old* adult* or old* individual* or old* men or old* man or old* patient* or old* people or old* person* or old* population* or old* woman or old* women or oldest old).ti,ab,kf. |
| 21 | (("5#" or "6#" or "7#" or "8#" or "9#") adj3 year? adj3 (age* or old*)).ti,ab,kf. |
| 22 | or/19-21 |
| 23 | 18 and 22 |
| 24 | 23 not (animals not humans).sh. |
| 25 | limit 24 to (congress or consensus development conference or consensus development conference, nih or editorial or festschrift or guideline or historical article or interview or lecture or letter or "meta analysis" or news or newspaper article or personal narrative or portrait or "review" or "systematic review") |
| 26 | 24 not 25 |

Embase

('fat intake'/de OR 'fatty acid'/de OR 'essential fatty acid'/exp OR 'monounsaturated fatty acid'/de OR 'unsaturated fatty acid'/de OR 'trans fatty acid'/de OR 'omega 3 fatty acid'/de OR 'omega 6 fatty acid'/de OR 'daleuton'/de OR 'hexanoic acid derivative'/de OR 'octanoic acid derivative'/de OR 'decanoic acid'/de OR 'arachidic acid'/de OR 'lauric acid derivative'/de OR 'myristic acid'/de OR 'oleic acid'/de OR 'palmitic acid'/de OR 'vegetable oil'/de OR 'stearic acid derivative'/de OR 'docosahexaenoic acid'/de OR 'icosapentaenoic acid'/de OR 'conjugated linoleic acid'/de OR (('fatty acid*' NEAR/3 ('long chain' OR 'medium chain' OR n3 OR 'n-3' OR n6 OR 'n-6' OR 'odd chain' OR 'omega-3' OR 'omega-6')):ti,ab,kw) OR ('fatty acid*':ti,ab,kw AND (consum*:ti,ab,kw OR diet*:ti,ab,kw OR intake:ti,ab,kw OR ingest*:ti,ab,kw)) OR 'butter'/de OR 'dressing'/de OR 'ghee'/de OR 'lard'/de OR 'margarine'/de OR butter:ti,ab,kw OR 'dietary fat*':ti,ab,kw OR dressing*:ti,ab,kw OR 'fat intake':ti,ab,kw OR ghee:ti,ab,kw OR lard:ti,ab,kw OR margarin:ti,ab,kw OR ((('fatty acid*' OR fat OR fats) NEAR/3 (conjugated OR essential OR monounsaturat* OR polyunsaturat* OR saturat* OR trans OR unsaturat*)):ti,ab,kw) OR dha:ti,ab,kw OR epa:ti,ab,kw OR mufa:ti,ab,kw OR 'lc pufa':ti,ab,kw OR pufa:ti,ab,kw OR sfa:ti,ab,kw OR tfa:ti,ab,kw OR transfat*:ti,ab,kw OR 'corn oil'/de OR ‘cotton seed oil’/de OR ‘cooking oil’/de OR ‘edible oil’/de OR ‘olive oil’/exp OR ‘vegetable oil’/de OR ‘safflower oil’/de OR ‘sesame seed oil’/de OR ‘soybean oil’/de OR (oil$:ti,ab,kw AND (consum*****:ti,ab,kw OR diet*:ti,ab,kw OR intake:ti,ab,kw OR ingest*:ti,ab,kw) OR ('8,11,14-eicosatrienoic acid':ti,ab,kw OR 'alpha-linolenic':ti,ab,kw) AND acid:ti,ab,kw OR 'arachidic acid':ti,ab,kw OR 'arachidonic acid':ti,ab,kw OR 'behenic acid':ti,ab,kw OR caproates:ti,ab,kw OR 'capric acids':ti,ab,kw OR caprylates:ti,ab,kw OR 'decanoic acids':ti,ab,kw OR 'cervonic acid':ti,ab,kw OR 'cerotic acid':ti,ab,kw OR 'dihomo-gamma-linolenic acid':ti,ab,kw OR 'docosahexaenoic acid':ti,ab,kw OR 'docosanoic acid':ti,ab,kw OR 'eicosanoic acids':ti,ab,kw OR 'eicosapentaneoic acid':ti,ab,kw OR 'elaic acid':ti,ab,kw OR 'gamma-linolenic acid':ti,ab,kw OR 'gondoic acid':ti,ab,kw OR 'heptadecanoic acid':ti,ab,kw OR 'hexacosanoic acid':ti,ab,kw OR 'icosapentaenoic acid':ti,ab,kw OR 'delauric acids':ti,ab,kw OR 'lignoceric acid':ti,ab,kw OR 'linoleic acid':ti,ab,kw OR 'linolelaidic acid':ti,ab,kw OR 'margaric acid':ti,ab,kw OR 'montanic acid':ti,ab,kw OR 'myristic acid':ti,ab,kw OR 'octanoic acid':ti,ab,kw OR 'oleic acid':ti,ab,kw OR 'palmitoleic acid':ti,ab,kw OR 'palmitole* acid':ti,ab,kw OR 'palmitic acid':ti,ab,kw OR 'paullinic acid':ti,ab,kw OR 'pentadecanoic acid':ti,ab,kw OR 'pentadecylic acid':ti,ab,kw OR 'stearic acid?':ti,ab,kw OR 'stearidonic acid':ti,ab,kw))

AND

('cognitive defect'/exp OR alzheimer*:ti,ab,kw OR aphasia:ti,ab,kw OR binswanger*:ti,ab,kw OR cadasil:ti,ab,kw OR cadasilm:ti,ab,kw OR dementia*:ti,ab,kw OR 'frontotemporal lobar degeneration':ti,ab,kw OR ((cognit* NEAR/2 (decline OR disorder* OR dysfunction* OR impair*)):ti,ab,kw) OR ((subcortical NEAR/3 (encephalopathy OR leukoencephalopathy)):ti,ab,kw))
AND

('middle aged'/de OR 'aged'/exp OR aged:ti,ab,kw OR aging:ti,ab,kw OR ageing:ti,ab,kw OR elder*:ti,ab,kw OR geriatr*:ti,ab,kw OR sexagenarian*:ti,ab,kw OR septuagenarian*:ti,ab,kw OR octogenarian*:ti,ab,kw OR nonagenarian*:ti,ab,kw OR centenarian*:ti,ab,kw OR 'middle* age*':ti,ab,kw OR 'old age':ti,ab,kw OR 'old* adult*':ti,ab,kw OR 'old* individual*':ti,ab,kw OR 'old* men':ti,ab,kw OR 'old* man':ti,ab,kw OR 'old* patient*':ti,ab,kw OR 'old* people':ti,ab,kw OR 'old* person*':ti,ab,kw OR 'old* population*':ti,ab,kw OR 'old* woman':ti,ab,kw OR 'old* women':ti,ab,kw OR 'oldest old':ti,ab,kw OR ((('5#' OR '6#' OR '7#' OR '8#' OR '9#') NEAR/3 year? NEAR/3 (age* OR old*)):ti,ab,kw))

NOT ([animals]/lim NOT [humans]/lim) AND ([article]/lim OR [article in press]/lim OR [erratum]/lim)

Cochrane Library

| **ID** | **Search** |
| --- | --- |
| #1 | MeSH descriptor: [Dietary Fats] explode all trees |
| #2 | MeSH descriptor: [Fatty Acids] this term only |
| #3 | MeSH descriptor: [Fatty Acids, Essential] this term only |
| #4 | MeSH descriptor: [Fatty Acids, Monounsaturated] this term only |
| #5 | MeSH descriptor: [Fatty Acids, Unsaturated] this term only |
| #6 | MeSH descriptor: [Trans Fatty Acids] this term only |
| #7 | MeSH descriptor: [Fatty Acids, Omega-3] explode all trees |
| #8 | MeSH descriptor: [Fatty Acids, Omega-6] explode all trees |
| #9 | MeSH descriptor: [8,11,14-Eicosatrienoic Acid] this term only |
| #10 | MeSH descriptor: [Arachidonic Acids] this term only |
| #11 | MeSH descriptor: [Caproates] this term only |
| #12 | MeSH descriptor: [Caprylates] this term only |
| #13 | MeSH descriptor: [Decanoic Acids] this term only |
| #14 | MeSH descriptor: [Eicosanoic Acids] this term only |
| #15 | MeSH descriptor: [Lauric Acids] this term only |
| #16 | MeSH descriptor: [Myristic Acid] this term only |
| #17 | MeSH descriptor: [Oleic Acid] this term only |
| #18 | MeSH descriptor: [Palmitic Acid] this term only |
| #19 | MeSH descriptor: [Plant Oils] this term only |
| #20 | MeSH descriptor: [Stearic Acids] this term only |
| #21 | ((fatty NEXT acid*) NEAR/3 ("long chain" or "medium chain" or n3 or "n-3" or n6 or "n-6" or "odd chain" or "omega-3" or "omega-6")):ti,ab,kw |
| #22 | ((fatty NEXT acid*) and (consum* or diet* or intake or ingest*)):ti,ab,kw |
| #23 | (butter or (dietary NEXT fat*) or dressing* or ghee or lard or margarine):ti,ab,kw |
| #24 | (((fatty NEXT acid*) or fat or fats) NEAR/3 (conjugated or essential or monounsaturat* or polyunsaturat* or saturat* or trans or unsaturat*)):ti,ab,kw |
| #25 | (dha or epa or mufa or "lc-pufa" or pufa or sfa or tfa):ti,ab,kw |
| #26 | (oil? and (consum* or diet* or intake or ingest)):ti,ab,kw |
| #27 | ("8,11,14-Eicosatrienoic acid" or "alpha-Linolenic acid" or "arachidic acid" or "arachidonic acid" or "behenic acid" or caproates or "capric acids" or caprylates or "decanoic acids" or "cervonic acid" or "cerotic acid" or "dihomo-gamma-linolenic acid" or "docosahexaenoic acid" or "docosanoic acid" or "eicosanoic acids" or "eicosapentaneoic acid" or "elaic acid" or "gamma-linolenic acid" or "gondoic acid" or "heptadecanoic acid" or "hexacosanoic acid" or "icosapentaenoic acid" or "lauric acids" or "lignoceric acid" or "linoleic acid" or "linolelaidic acid" or "margaric acid" or "montanic acid" or "myristic acid" or "octanoic acid" or "oleic acid" or (palmitole* NEXT acid) or "palmitic acid" or "paullinic acid" or "pentadecanoic acid" or "pentadecylic acid" or "stearic acids" or "stearidonic acid"):ti,ab,kw |
| #28 | #1 or #2 or #3 or #4 or #5 or #6 or #7 or #8 or #9 or #10 or #11 or #12 or #13 or #14 or #15 or #16 or #17 or #18 or #19 or #20 or #21 or #22 or #23 or #24 or #25 or #26 or #27 |
| #29 | MeSH descriptor: [Cognition Disorders] this term only |
| #30 | MeSH descriptor: [Cognitive Dysfunction] this term only |
| #31 | MeSH descriptor: [Dementia] explode all trees |
| #32 | (Alzheimer* or aphasia or Binswanger* or CADASIL or CADASILM or dementia* or "frontotemporal lobar degeneration"):ti,ab,kw |
| #33 | (cognit* NEAR/2 (decline or disorder* or dysfunction* or impair*)):ti,ab,kw |
| #34 | (subcortical NEAR/3 (encephalopathy or leukoencephalopathy)):ti,ab,kw |
| #35 | #29 or #30 or #31 or #32 or #33 or #34 |
| #36 | MeSH descriptor: [Middle Aged] this term only |
| #37 | MeSH descriptor: [Aged] explode all trees |
| #38 | (aged or aging or ageing or elder* or geriatr* or sexagenarian* or septuagenarian* or octogenarian* or nonagenarian* or centenarian* or (middle* NEXT age*) or "old age" or (old* NEXT adult*) or (old* NEXT individual*) or (old* NEXT men) or (old* NEXT man) or (old* NEXT patient*) or (old* NEXT people) or (old* NEXT person*) or (old* NEXT population*) or (old* NEXT woman) or (old* NEXT women) or "oldest old"):ti,ab,kw |
| #39 | (("50" OR "51" OR "52" OR "53" OR "54" OR "55" OR "56" OR "57" OR "58" OR "59" OR "60" OR "61" OR "62" OR "63" OR "64" OR "65" OR "66" OR "67" OR "68" OR "69" OR "70" OR "71" OR "72" OR "73" OR "74" OR "75" OR "76" OR "77" OR "78" OR "79" OR "80" OR "81" OR "82" OR "83" OR "84" OR "85" OR "86" OR "87" OR "88" OR "89" OR "90" OR "91" OR "92" OR "93" OR "94" OR "95" OR "96" OR "97" OR "98" OR "99") NEAR/3 year* NEAR/3 (age* OR old*)):ti,ab,kw |
| #40 | #36 or #37 or #38 or #39 |
| #41 | #28 AND #35 AND #40 |

Scopus

( ( TITLE-ABS-KEY ( ( "fatty acid*" W/2 ( "long chain" OR "medium chain" OR n3 OR "n-3" OR n6 OR "n-6" OR "odd chain" OR "omega-3" OR "omega-6" ) ) ) ) OR ( TITLE-ABS-KEY ( "fatty acid*" AND ( consum* OR diet* OR intake OR ingest* ) ) ) OR ( TITLE-ABS-KEY ( butter OR "dietary fat*" OR dressing* OR ghee OR lard OR margarine ) ) OR ( TITLE-ABS-KEY ( ( "fatty acid*" OR fat OR fats ) W/2 ( conjugated OR essential OR monounsaturat* OR polyunsaturat* OR saturat* OR trans OR unsaturat* ) ) ) OR ( TITLE-ABS-KEY ( dha OR epa OR mufa OR "lc-pufa" OR pufa OR sfa OR tfa OR transfat* ) ) OR ( TITLE-ABS-KEY ( oil* AND ( consum* OR diet* OR intake OR ingest* ) ) ) OR ( TITLE-ABS-KEY ( "8,11,14-Eicosatrienoic acid" OR "alpha-Linolenic acid" OR "arachidic acid" OR "arachidonic acid" OR "behenic acid" OR caproates OR "capric acids" OR caprylates OR "decanoic acids" OR "cervonic acid" OR "cerotic acid" OR "dihomo-gamma-linolenic acid" OR "docosahexaenoic acid" OR "docosanoic acid" OR "eicosanoic acids" OR "eicosapentaneoic acid" OR "elaic acid" OR "gamma-linolenic acid" OR "gondoic acid" OR "heptadecanoic acid" OR "hexacosanoic acid" OR "icosapentaenoic acid" OR "lauric acids" OR "lignoceric acid" OR "linoleic acid" OR "linolelaidic acid" OR "margaric acid" OR "montanic acid" OR "myristic acid" OR "octanoic acid" OR "oleic acid" OR "palmitoleic acid" OR "palmitole* acid" OR "palmitic acid" OR "paullinic acid" OR "pentadecanoic acid" OR "pentadecylic acid" OR "stearic acids" OR "stearidonic acid" ) ) ) AND ( ( TITLE-ABS-KEY ( alzheimer* OR aphasia OR binswanger* OR cadasil OR cadasilm OR dementia* OR "frontotemporal lobar degeneration" ) ) OR ( TITLE-ABS-KEY ( cognit* W/1 ( decline OR disorder* OR dysfunction* OR impair* ) ) ) OR ( TITLE-ABS-KEY ( subcortical W/2 ( encephalopathy OR leukoencephalopathy ) ) ) ) AND ( ( TITLE-ABS-KEY ( aged OR aging OR ageing OR elder* OR geriatr* OR sexagenarian* OR septuagenarian* OR octogenarian* OR nonagenarian* OR centenarian* OR "middle* age*" OR "old age" OR "old* adult*" OR "old* individual*" OR "old* men" OR "old* man" OR "old* patient*" OR "old* people" OR "old* person*" OR "old* population*" OR "old* woman" OR "old* women" OR "oldest old" ) ) OR ( TITLE-ABS-KEY ( ( "50" OR "51" OR "52" OR "53" OR "54" OR "55" OR "56" OR "57" OR "58" OR "59" OR "60" OR "61" OR "62" OR "63" OR "64" OR "65" OR "66" OR "67" OR "68" OR "69" OR "70" OR "71" OR "72" OR "73" OR "74" OR "75" OR "76" OR "77" OR "78" OR "79" OR "80" OR "81" OR "82" OR "83" OR "84" OR "85" OR "86" OR "87" OR "88" OR "89" OR "90" OR "91" OR "92" OR "93" OR "94" OR "95" OR "96" OR "97" OR "98" OR "99" ) W/2 year* W/2 ( age* OR old* ) ) ) ) AND ( EXCLUDE ( DOCTYPE , "re" ) OR EXCLUDE ( DOCTYPE , "ch" ) OR EXCLUDE ( DOCTYPE , "cp" ) OR EXCLUDE ( DOCTYPE , "ed" ) OR EXCLUDE ( DOCTYPE , "le" ) OR EXCLUDE ( DOCTYPE , "no" ) OR EXCLUDE ( DOCTYPE , "bk" ) OR EXCLUDE ( DOCTYPE , "cr" ) )
